# CalliSpheres^®^ microsphere transarterial chemoembolization combined with ^125^I brachytherapy for patients with non–small‐cell lung cancer liver metastases

**DOI:** 10.3389/fonc.2022.882061

**Published:** 2022-08-12

**Authors:** Guangsheng Zhao, Song Liu, Ying Liu, Xiang Li, Guangji Yu, Yuewei Zhang, Jie Bian, Jianlin Wu, Jun Zhou, Fei Gao

**Affiliations:** ^1^ Cancer Interventional Center, Affiliated Zhongshan Hospital of Dalian University, Dalian, China; ^2^ Cancer Interventional Center, Linyi Cancer Hospital, Linyi, China; ^3^ Hepatobiliary and Pancreatic Center, Beijing Tsinghua Changgung Hospital, Beijing, China; ^4^ Department of Radiology, The Second Affiliated Hospital of Dalian Medical University, Dalian, China; ^5^ Department of Radiology, Affiliated Zhongshan Hospital of Dalian University, Dalian, China; ^6^ Cancer Interventional Center, The Second Affiliated Hospital of Dalian Medical University, Dalian, China

**Keywords:** non–small‐cell lung cancer liver metastases, CalliSpheres^®^ microsphere transarterial chemoembolization plus ^125^I brachytherapy, treatment response, survival, adverse events

## Abstract

**Objective:**

Poor prognosis and limited treatments of liver metastases from non–small‐cell lung cancer (NSCLC) after radical surgery are critical issues. The current study aimed to evaluate the efficacy and safety of CalliSpheres^®^ microsphere transarterial chemoembolization (CSM-TACE) plus ^125^I brachytherapy in these patients.

**Methods:**

A total of 23 patients with liver metastases from NSCLC after radical surgery were included. All patients received CSM-TACE 1–3 times, then ^125^I brachytherapy was carried out following the last CSM-TACE. Complete response (CR), objective response rate (ORR), disease control rate (DCR), survival, and adverse events were evaluated.

**Results:**

CR, ORR and DCR were 43.5%, 87.0%, and 100%, respectively, at three months; furthermore, they were 78.3%, 100%, and 100% accordingly at six months. Moreover, most European Organization for Research and Treatment of Cancer Quality of Life Questionnaire-Core 30 (QLQ-C30) subscales of functions (including physical and emotional function) and symptoms (including pain, nausea, and vomiting) were generally improved at three months (all *P* < 0.05). Furthermore, median progression-free survival (PFS) was 14.0 [95% confidence interval (CI): 10.4–17.6] months, with a 1-year PFS rate of 62.9%, but the 2-year PFS rate was not reached. Moreover, the median overall survival (OS) was 22.0 (95% CI: 16.8–27.2) months, with a 1-year OS rate of 91.3% and a 2-year OS rate of 43.5%. Additionally, the main adverse events included fever (100%), pain (65.2%), liver function impairment (65.2%), fatigue (56.5%), and nausea and vomiting (52.2%), which were all categorized as grade 1–2.

**Conclusion:**

CSM-TACE plus ^125^I brachytherapy is effective and safe in patients with liver metastases from NSCLC after radical surgery, providing a potentially optimal option in these patients.

## 1 Introduction

Non–small‐cell lung cancer (NSCLC) is the second most common cancer worldwide and one of the leading causes of cancerous death in China (1, 2). Over the decades, with the advancement in diagnostic techniques and identification of risk factors, increasing NSCLC patients are diagnosed at an early stage and have the opportunity to receive surgical resection (1, 2). However, a proportion of NSCLC patients after radical surgery may develop tumor metastases, including the liver as one of the most common metastatic sites (3–5). Currently, surgery and chemotherapy are recommended for patients with liver metastases from NSCLC after radical surgery, while the therapeutic effect is unfavorable (3, 6, 7). Considering that liver metastasis from NSCLC after radical surgery is still a critical challenge, the exploration of more effective and safe treatments is urgent.

In the past years, conventional transarterial chemoembolization (cTACE) has been viewed as a crucial treatment for liver metastases, while its therapeutic effect remains unsatisfactory (8–10). Recently, drug-eluting bead (DEB)-TACE has drawn a lot of attention in liver metastases, among which DEB-TACE using CalliSpheres^®^ microspheres (the first self-developed DEB in China) exhibits satisfying efficacy and tolerable safety not only in primary liver cancer but also in liver metastases (10–13). For instance, CalliSpheres^®^ microsphere (CSM)-TACE shows acceptable survival benefits and tolerable safety in colorectal cancer liver metastases (13). Moreover, CSM-TACE presents a favorable treatment response and manageable adverse events in gastric cancer liver metastases (10). However, the efficacy and safety of CSM-TACE in liver metastases from NSCLC after radical surgery are unclear.

It has been reported that ^125^I seeds can be accurately implanted into the tumor under the assistance of a three-dimensional (3D) template, which is able to continuously release a low dose of radiation on tumor tissue with minimal invasion and consequently kill tumor cells, leading to favorable efficacy and satisfactory safety in several cancers, including liver metastases (14–17). Interestingly, it has been reported that the combination of DEB-TACE and ^125^I brachytherapy exerts satisfying efficacy in primary liver cancer (18, 19). However, the data about the efficacy and safety of their combination in liver metastases are obscure.

Therefore, the current study aimed to explore the efficacy and safety of integrated interventional therapy of CSM-TACE combined with ^125^I brachytherapy in patients with liver metastases from NSCLC after radical surgery.

## 2 Methods

### 2.1 Patients

From May 2018 to May 2019, the current prospective study consecutively included 23 patients with liver metastases from NSCLC after surgical resection who received integrated interventional therapy of CSM-TACE combined with ^125^I brachytherapy after the failure of second or above-line chemotherapy. The patients were enrolled from four cancer centers in China and scheduled for integrated interventional therapy of CSM-TACE combined with ^125^I brachytherapy. The inclusion criteria were as follows: 1) pathologically or imageologically confirmed NSCLC; 2) suffered from liver metastases after radical surgery for NSCLC, which were confirmed by puncture biopsy or imageological examinations; 3) age within 18–85 years; 4) failure of second or above-line chemotherapy for liver metastases; 5) Child–Pugh class A–B; 6) liver tumor size less than two-thirds of the liver; 7) life expectancy >6 months. The exclusion criteria were as follows: 1) allergy to drugs or materials used in the study; 2) Child–Pugh class C; 3) Eastern Cooperative Oncology Group performance status (ECOG PS) score >2; 4) unable to complete the CSM-TACE combined with ^125^I brachytherapy. All patients gave written informed consent and participated in the study voluntarily. The study protocol was approved by the ethics committees of the following: 1) Affiliated Zhongshan Hospital of Dalian University; 2) Linyi Cancer Hospital; 3) Beijing Tsinghua Changgung Hospital; 4) The Second Affiliated Hospital of Dalian Medical University.

### 2.2 Treatment

All patients received CSM-TACE 1–3 times. After 1 month of the last CSM-TACE treatment, the lesions were stabilized (the active lesions were reduced, and there was no tumor-feeding artery), and ^125^I brachytherapy was performed. All patients did not receive other treatments in parallel in addition to CSM-TACE and ^125^I.

#### CalliSpheres^®^ microsphere transarterial chemoembolization Procedures

The specific methods were the same as those described in our previous studies (12, 13). Different from previous studies, this study used CSM with an inner diameter ranging from 100 to 300 μm to load 20-mg lobaplatin injection. The prepared drug-loaded microspheres were slowly infused into the tumor-feeding artery and the regional arteries at the edge of the tumor. The gelatin sponge particles (150~350 μm) were used as supplementary embolism materials when the tumor size was larger than 5 cm, or a bottle of drug-loaded CSM was not enough to completely embolize the tumor. Criteria for the completion of embolization were that the blood flow in the blood-supplying arteries of the tumor was stagnant, and tumor staining completely disappeared in the angiography.

#### ^125^I Brachytherapy

The ^125^I seed implantation was performed as described in our previous study (15). In brief, domestic radioactive ^125^I seeds with a half-life of 60.2 days, an activity of 0.6–0.8 mCi (1 Ci = 3.7 × l0^10^ Bq), and a γ-ray energy of 27–35 keV were applied in this study. The prescribed dose (PD) in the planned target area was 110–180 Gy. The main areas of ^125^I seed implantation were tumor margin and residual active area. After the installation of the positioning navigation instrument and 3D template, the insertion position and angle of the needle were controlled, and the needle insertion channel was established according to a preoperative plan. Starting from the central plane of the tumor, the implanted needles were arranged in layers, with a lateral margin of 1 cm, a depth of 0.5 cm from the distal edge, and a distance of more than 1 cm from the skin. Meanwhile, intraoperative plan modification and target dose optimization were conducted as well. In addition, the whole procedure also included the formulation of brachytherapy treatment planning system (BTPS) preoperative plan and postoperative dosimetry verification of BTPS.

After treatment, the results of CSM-TACE were comprehensively evaluated and the decision to reintervene was made based on the results of the imaging review. If there were localized recurrent lesions in the liver, the protocol was repeated in conjunction with the patient’s physical status, etc.

### 2.3 Outcome assessment

Using enhanced magnetic resonance imaging or computed tomography (CT), the treatment response was evaluated at 3 and 6 months after integrated interventional therapy according to the modified response evaluation criteria in solid tumors (mRECIST) assessment for hepatocellular carcinoma (20). The general health status of patients was assessed before and 3 months after integrated interventional therapy using the European Organization for Research and Treatment of Cancer Quality of Life Questionnaire-Core 30 (QLQ-C30) scale. The adverse events of CSM-TACE and ^125^I were evaluated and graded referring to the National Cancer Institute Common Terminology Criteria for Adverse Events (CTCAE, version 4.0, available at: http://www.eortc.be/services/doc/ctc/CTCAE_4.03_2010-06-14_QuickReference_5x7.pdf) (myelosuppression and liver function impairment were evaluated per CTCAE, version 3.0). Moreover, the major complications of ^125^I were also recorded. In addition, all patients were consecutively followed up until May 2021, and progression-free survival (PFS) and overall survival (OS) were calculated. PFS was defined as the duration between the initiation of CSM-TACE and disease progression or patient’s death, whichever occurred first. OS was defined as the duration between the initiation of CSM-TACE and the death of the patient. Patients who did not experience a PFS or OS event at analysis were recorded at their last date of disease assessment.

### 2.4 Statistical analysis

Data processing was completed by SPSS 20.0 software (IBM Corp., Armonk, NY, USA). Data were described as mean with standard deviation (SD), median with interquartile range (IQR), or count with percentages, as appropriate. A comparison was made using Wilcoxon signed rank test or log-rank test. PFS and OS were displayed by Kaplan–Meier curves. A *P* value <0.05 indicated statistical significance.

## 3 Results

### 3.1 Clinical characteristics

A total of 23 patients were included in the current research. The mean age was 55.2 ± 10.7 years. Furthermore, there were 17 (73.9%) men and 6 (26.1%) women. Regarding histological types, 19 (82.6%) patients possessed squamous cell carcinoma and 4 (17.4%) patients had adenocarcinoma. With respect to the ECOG PS score, 18 (78.3%) patients had a score of 0 and 5 (21.7%) patients had a score of 1. As for the number of liver metastases, 10 (43.5%), 7 (30.4%), and 6 (26.1%) patients had 1–3, 4–5, and ≥6 liver metastases, respectively. Furthermore, 12 (52.2%) patients presented extrahepatic metastases and 11 (47.8%) patients did not. Regarding the largest diameter of liver metastases, 5 (21.7%), 7 (30.4%), and 11 (47.8%) patients possessed ≤3-, 3–5-, and >5-cm liver metastases accordingly. Moreover, 9 (39.1%) and 14 (60.9%) patients previously received second-line and third or above-line systemic treatments, respectively (**Table 1**).

**Table 1:** Clinical characteristics.

Items	Patients (N = 23)
Age (years), mean ± SD	55.2 ± 10.7
Gender, No. (%)	
Men	17 (73.9)
Women	6 (26.1)
Histological type, No. (%)	
SCC	19 (82.6)
ADC	4 (17.4)
ECOG score, No. (%)	
0	18 (78.3)
1	5 (21.7)
Child–Pugh class, No. (%)	
A	19 (82.6)
B	4 (17.4)
Number of liver metastases, No. (%)	
1∼3	10 (43.5)
4∼5	7 (30.4)
≥6	6 (26.1)
Extrahepatic metastasis, No. (%)	
Present	12 (52.2)
Absent	11 (47.8)
Largest diameter of liver metastases, No. (%)	
≤3 cm	5 (21.7)
3∼5 cm	7 (30.4)
>5 cm	11 (47.8)
Previous systemic treatment, No. (%)	
Second line	9 (39.1)
Third or above lines	14 (60.9)

SD, standard deviation; SCC, squamous cell carcinoma; ADC, adenocarcinoma; ECOG, Eastern Cooperative Oncology Group.

### 3.2 Clinical response at 3 and 6 months

The included 23 patients received a total of 47 cycles of CSM-TACE treatment, with an average of 2.04 ± 0.50 cycles. Furthermore, a plain CT scan showed that tumors presented uniformly distributed low-density necrosis and partial tumors illustrated necrosis in alveolate form on 4 days after CSM-TACE. Then, ^125^I brachytherapy was conducted in 37 tumors of 23 patients. Furthermore, the number of ^125^I seeds ranged from 53 to 119, with an average number of 61.

Using enhanced magnetic resonance imaging or CT, treatment response was evaluated after 3 and 6 months among patients treated with integrated interventional therapy. The data showed that after 3 months, 10 (43.5%), 10 (43.5%), 3 (13.0%), and 0 (0.0%) patients achieved complete response (CR), partial response (PR), stable disease (SD), and progressive disease (PD), respectively. Meanwhile, objective response rate (ORR) and disease control rate (DCR) were 87.0% and 100.0% accordingly. After 6 months, 18 (78.3%), 5 (21.7%), 0 (0.0%), and 0 (0.0%) patients realized CR, PR, SD, and PD, respectively. Furthermore, both ORR and DCR were 100% (**Table 2**).

**Table 2:** Clinical response at 3 and 6 months after treatment.

Time points	CR	PR	SD	PD	ORR	DCR
3 months	10 (43.5)	10 (43.5)	3 (13.0)	0 (0.0)	20 (87.0)	23 (100.0)
6 months	18 (78.3)	5 (21.7)	0 (0.0)	0 (0.0)	23 (100.0)	23 (100.0)

CR, complete response; PR, partial response; SD, stable disease; PD, progressive disease; ORR, objective response rate; DCR, disease control rate.

### 3.3 Quality of life before and at 3 months after treatment

QLQ-C30 score was used to assess the quality of life of patients. The data presented that the score of physical function, emotional function, and general health status were all elevated at 3 months after treatment (all *P* < 0.05). Furthermore, the score of pain, nausea and vomiting, insomnia, and loss of appetite were all decreased 3 months after treatment (all *P* < 0.05) (**Table 3**).

**Table 3:** QLQ-C30 score before and at 3 months after treatment.

Items	QLQ-C30 score, median (IQR)		*P* value
	Before treatment	3 months after treatment	
Physical function	80.6 (52.4-85.7)	85.6 (85.6-96.4)	0.016
Role function	66.7 (66.5-99.5)	90.3 (65.5-100)	0.165
Cognitive function	83.3 (67.7-100)	90.7 (67.7-100)	0.090
Emotional function	84.3 (68.7-100)	95.7 (83.3-100)	0.020
Social function	66.5 (66.5-100)	100.0 (83.3-100)	0.369
General health status	57.6 (50.0-66.5)	65.7 (65.6-82.3)	0.017
Fatigue	45.1 (34.0-56.0)	12.9 (11.9-34.2)	0.087
Pain	16.5 (0.0-16.5)	0.0 (0.0-16.5)	0.010
Nausea and vomiting	0.0 (0.0-16.5)	0.0 (0.0-0.0)	0.023
Shortness of breath	33.3 (33.3-66.7)	0.0 (0.0-33.3)	0.203
Insomnia	33.3 (0.0-67.0)	0.0 (0.0-33.3)	0.020
Loss of appetite	36.5 (0.0-66.7)	35.3 (0.0-35.3)	0.006
Constipation	0.0 (0.0-16.7)	0.0 (0.0-0.0)	0.300
Diarrhea	0.0 (0.0-16.7)	0.0 (0.0-0.0)	0.900
Financial difficulty	0.0 (0.0-33.3)	0.0 (0.0-16.7)	0.164

QLQ-C30, Quality of Life Questionnaire-Core 30; IQR, interquartile range.

### 3.4 Progression-free survival and overall survival evaluation

**Figure 1:**
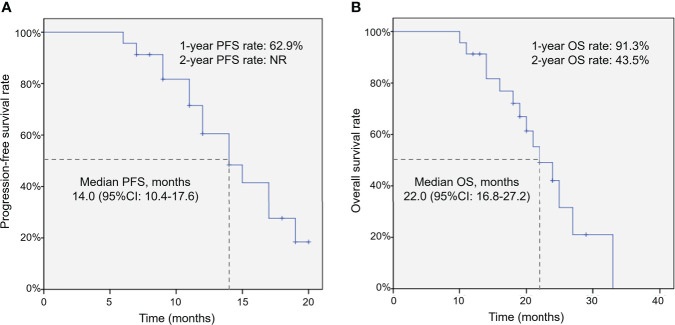
Survival of patients with liver metastases from NSCLC after integrated interventional therapy. PFS **(A)** and OS **(B)** among patients after integrated interventional therapy of CSM-TACE combined with ^125^I brachytherapy. PFS, progression-free survival; OS, overall survival; NSCLC, non–small‐cell lung cancer; CSM-TACE, CalliSpheres^®^ microsphere transarterial chemoembolization; CI, confidence interval.

After integrated interventional therapy, the median PFS was 14.0 [95% confidence interval (CI): 10.4–17.6] months; meanwhile, the 1-year PFS rate was 62.9% but a 2-year PFS rate was not reached (NR) (**Figure 1A**). Additionally, the median OS was 22.0 (95% CI: 16.8–27.2) months; moreover, the 1- and 2-year OS rates were 91.3% and 43.5%, respectively (**Figure 1B**).

In addition, extrahepatic metastasis was not correlated with PFS (*P* = 0.324) (**Supplementary Figure S1A**) or OS (*P* = 0.484) (**Supplementary Figure S1B**); meanwhile, tumor size was not related to PFS (*P* = 0.494) (**Supplementary Figure S1C**) or OS (*P* = 0.436) (**Supplementary Figure S1D**).

### 3.5 Adverse events

The adverse events included fever (100%), pain (65.2%), liver function impairment (65.2%), fatigue (56.5%), nausea and/or vomiting (52.2%), myelosuppression (4.3%), and biloma (4.3%), which all belonged to grade 1–2. Meanwhile, no grade 3–4 adverse events were found.

The adverse events related to CSM-TACE included fever, pain, nausea and vomiting, fatigue, liver function impairment, and biloma, which were effectively managed after medical treatment; moreover, there were no severe complications. In addition, the main adverse events related to ^125^I brachytherapy were grade 1 hepatic pain and liver function impairment (**Table 4**). Moreover, complications of ^125^I brachytherapy included manageable intrahepatic bile duct injury, vascular injury, and pneumothorax (**Supplementary Table S1**).

**Table 4:** Adverse events.

Items	Total	Grade 1	Grade 2	Grade 3	Grade 4
Adverse events of CMS-TACE					
Fever, No. (%)	23 (100.0)	14 (60.9)	9 (39.1)	0 (0.0)	0 (0.0)
Pain, No. (%)	15 (65.2)	11 (47.8)	4 (17.4)	0 (0.0)	0 (0.0)
Liver function impairment, No. (%)	15 (65.2)	11 (47.8)	4 (17.4)	0 (0.0)	0 (0.0)
Fatigue, No. (%)	13 (56.5)	11 (47.8)	2 (8.7)	0 (0.0)	0 (0.0)
Nausea and/or vomiting, No. (%)	12 (52.2)	9 (39.1)	3 (13.0)	0 (0.0)	0 (0.0)
Myelosuppression, No. (%)	1 (4.3)	1 (4.3)	0 (0.0)	0 (0.0)	0 (0.0)
Biloma, No. (%)	1 (4.3)	1 (4.3)	0 (0.0)	0 (0.0)	0 (0.0)
**Adverse events of ^125^I**					
Hepatic pain, No. (%)	4 (17.4)	4 (17.4)	0 (0.0)	0 (0.0)	0 (0.0)
Liver function impairment, No. (%)	2 (8.7)	2 (8.7)	0 (0.0)	0 (0.0)	0 (0.0)

CSM-TACE, CalliSpheres^®^ microsphere transarterial chemoembolization.

### 3.6 Presentation of a typical case

**Figure 2:**
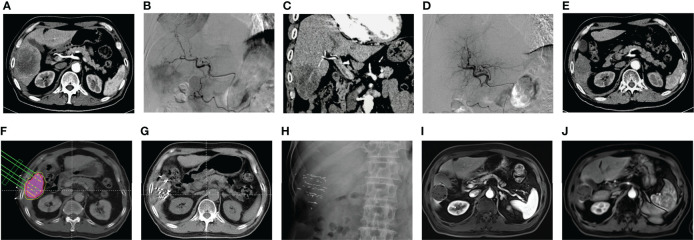
A typical case. Enhanced CT **(A)**, intraoperative DSA **(B)**, and DSA 3D reconstruction **(C)** focusing on liver metastases before CSM-TACE. DSA focusing on liver metastases after CSM-TACE **(D)**. Enhanced CT focusing on liver metastases 1 month after the third round of CSM-TACE **(E)**. Simulation plan before ^125^I brachytherapy **(F)**. Intraoperative CT focusing on liver metastases immediately after ^125^I brachytherapy **(G)**. Abdominal X-ray focusing on liver metastases 3 days after ^125^I brachytherapy **(H)**. Enhanced MRI focusing on liver metastases 1 year **(I)** and 2 years **(J)** after integrated interventional therapy. CSM-TACE, CalliSpheres^®^ microsphere transarterial chemoembolization; CT, computed tomography; DSA, digital subtraction angiography; MRI, magnetic resonance imaging.

The process of a patient with liver metastases from NSCLC after radical surgery receiving CSM-TACE combined with ^125^I brachytherapy was presented in **Figure 2**, which was used as a typical case presentation. Before treatment, abdominal enhanced CT revealed an 8.7 cm × 6.1 cm tumor in the right hepatic lobe (**Figure 2A**), whose blood was supplied by hepatic artery branches indicated by intraoperative digital subtraction angiography (DSA) (**Figure 2B**); moreover, DSA 3D reconstruction presented hepatic space-occupying lesions (**Figure 2C**). Then, the patient underwent CSM-TACE. DSA after intervention presented that tumor staining completely disappeared and tumor blood vessels were truncated (**Figure 2D**). One month after the third round of CSM-TACE, enhanced CT revealed a reduction in size and an integral weak enhancement of the hepatic lesion (**Figure 2E**), indicating that CSM-TACE showed good treatment efficacy. Subsequently, a simulation plan was constructed before ^125^I seed implantation (**Figure 2F**). Then, CT after intervention showed that the result of intraoperative ^125^I seed implantation was similar to the simulation plan (**Figure 2G**). After 3 days of ^125^I brachytherapy, the abdominal X-ray illustrated that the ^125^I seed was distributed well (**Figure 2H**). After 1 year of integrated interventional therapy of CSM-TACE combined with ^125^I brachytherapy, the distribution of ^125^I seed was satisfying without obvious activity (**Figure 2I**). After 2 years of integrated interventional therapy, no obvious changes were found in tumor lesions (**Figure 2J**).

## 4 Discussion

Until now, the prognosis of patients with liver metastases from NSCLC after radical surgery is still unfavorable due to the limited efficacy of available treatments (such as chemotherapy and cTACE) (3, 21). Hence, the exploration of effective and safe therapy for patients with liver metastases from NSCLC after radical surgery has been a worldwide concern. In the current study, we disclosed that the integrated interventional therapy of CSM-TACE combined with ^125^I brachytherapy presented a favorable treatment response and survival as well as a satisfactory safety profile among patients with liver metastases from NSCLC after radical surgery.

Both CSM-TACE and ^125^I brachytherapy have been viewed as effective therapies for liver metastases (10, 13–15). It has been reported that CR, ORR, and DCR are 14.3%, 78.6%, and 90.5%, respectively, at 6 months after CSM-TACE in patients with liver metastases (13). In the current study, we found that among patients with liver metastases from NSCLC after radical surgery, CR, ORR, and DCR were 43.5%, 87.0%, and 100%, respectively, at 3 months; meanwhile, they were 78.3%, 100%, and 100% at 6 months after integrated interventional therapy. The possible explanations might be that 1) the efficacy of CSM-TACE was affected by tumor vascular condition, while ^125^I brachytherapy distributed inside or around the edges of the tumor as intentionally planned, thus ^125^I brachytherapy could complement the limitation of CSM-TACE, consequently the combination of the two treatments could present favorable efficacy (11, 22); 2) CSM-TACE was able to embolize tumor blood vessels and release a certain concentration of chemotherapeutic agents into them stably and continuously, which could realize arterial chemoembolization (12), while ^125^I brachytherapy served as radiotherapy, which could continuously irradiate tumor tissue and kill tumor and surrounding cells at different stages of fission (15). Thus, CSM-TACE was used to treat major lesions, while ^125^I brachytherapy could consolidate the effect of CSM-TACE by controlling surrounding lesions. The synergistic effect between CSM-TACE and ^125^I brachytherapy contributed to this combination treatment could exert superior efficacy among patients with liver metastases from NSCLC after radical surgery (13, 23).

The long-term prognosis of patients with liver metastases from NSCLC after radical surgery remains poor (5–7, 15). Moreover, a previous study presented a1-year OS rate of 81.0% in patients with liver metastases after CSM-TACE (13). Another study also proposes that 1-year OS rate is 73% among patients with liver metastases after ^125^I brachytherapy (16). In the current study, data showed that the median PFS was 14.0 (95% CI: 10.4–17.6) months with a 1-year PFS rate of 62.9%, and OS was 22.0 (95% CI: 16.8–27.2) months with a 1-year OS rate of 91.3%. Survival after integrated interventional therapy was relatively better than CSM-TACE or 125I brachytherapy alone (13, 16), which could be explained by that: 1) favorable treatment response resulted in satisfactory survival among patients; 2) CSM-TACE and ^125^I brachytherapy both served as locoregional treatments, whose effective time both could last for a long period, hence the combination of the two treatments would greatly prolong the survival of patients (14, 15, 24, 25).

Previous studies have reported that the adverse events related to CSM-TACE mainly include fever, pain, nausea, and vomiting, which are tolerable and manageable (11, 12, 25). Furthermore, the main adverse events related to ^125^I brachytherapy are tolerable and controllable intraoperative pneumothorax and skin ulcerations; moreover, the complications of ^125^I brachytherapy (including intrahepatic bile, vascular injury, and pneumothorax) are all manageable (14, 15, 22). In the current study, the main adverse events related to integrated interventional therapy were fever, pain, liver function impairment, and fatigue, which were all mild and manageable; meanwhile, there was no severe adverse event. The possible explanations might be that: 1) CSM-TACE sustainably released chemotherapy drug in the tumor, which avoided the spread of the chemotherapy drug into the systemic circulation, consequently minimizing the toxicity of the chemotherapy drug (11, 13); 2) ^125^I seed possessed a short radial distance, which could kill tumor cells without obvious injury for the neighboring cells, subsequently elevating the safety profile (26).

In the current study, the included patients all had failed systemic therapy: cisplatin, carboplatin, and other drugs that had been used in previous systemic therapy. Thus, we chose lobaplatin for TACE in consideration of drug resistance. In addition, our research center was the tumor center that carried out microparticle TACE treatment for liver cancer earlier. One of the main directions of clinical research in the early stage was to focus on absorbable gelatin sponge particles. The characteristics of gelatin sponge microspheres were suitable for regional tumor vascular embolization during TACE surgery, while other microspheres were not suitable for this method. Moreover, the absorbability of gelatin sponge microspheres could also reduce the occurrence of serious liver function damage, and the absorbability also provided the possibility for follow-up interventional therapy. Thus, the gelatin sponge particles (150~350 μm) were used as supplementary embolism materials in the current study. However, the current study had several limitations, as follows: 1) the sample size was relatively small; thus, a larger sample size study was still needed to further confirm the efficacy of CSM-TACE combined with ^125^I brachytherapy; 2) previous studies also propose that the antitumor immune and metabolic function of the liver still exists in patients with liver metastases; hence, the effect of CSM-TACE combined with ^125^I brachytherapy on these functions could be explored in the future (3, 27); 3) this study was single-arm; hence, randomized controlled trials could be conducted to further verify the efficacy and safety of CSM-TACE combined with ^125^I brachytherapy; 4) whether immunotherapy in addition to CSM-TACE combined with ^125^I brachytherapy could promote the management of patients with liver metastases from NSCLC could be explored in the forthcoming study.

In conclusion, integrated interventional therapy of CSM-TACE combined with ^125^I brachytherapy is effective and safe for patients with liver metastases from NSCLC after radical surgery, which provides a potentially optimal option for these patients.

## Data availability statement

The original contributions presented in the study are included in the article/**Supplementary Material**. Further inquiries can be directed to the corresponding authors.

## Ethics statement

The study protocol was approved by the Ethics Committee of Affiliated Zhongshan Hospital of Dalian University. The patients/participants provided their written informed consent to participate in this study.

## Author contributions

GZ, SL, YL, and JW made substantial contributions to the design of the present study. Data acquisition was performed by GZ, SL, YL, XL, GY, YZ, JB, JW, JZ, and FG. Data analysis was performed by GZ, SL, YL, JW, JZ, and FG. Data interpretation was performed by GZ, SL, YL, XL, GY, and YZ. GZ, SL, YL, JW, JZ, and FG critically revised the manuscript for important intellectual content. All authors approved the final version of the manuscript. All authors agree to be accountable for all aspects of the work in ensuring that questions related to the accuracy or integrity of the work are appropriately investigated and resolved.

## Conflict of interest

The authors declare that the research was conducted in the absence of any commercial or financial relationships that could be construed as a potential conflict of interest.

## Publisher’s note

All claims expressed in this article are solely those of the authors and do not necessarily represent those of their affiliated organizations, or those of the publisher, the editors and the reviewers. Any product that may be evaluated in this article, or claim that may be made by its manufacturer, is not guaranteed or endorsed by the publisher.

## Supplementary material

Supplementary Figure 1 Correlation of extrahepatic metastasis with PFS **(A)** and Correlation of extrahepatic metastasis and tumor size with survival. OS **(B)**; association of tumor size with PFS **(C)** and OS **(D)**. PFS, progression-free survival; OS, overall survival.
