# Supplementary figures and images for: CalliSpheres® microsphere transarterial chemoembolization combined with 125I brachytherapy for patients with non–small‐cell lung cancer liver metastases
Source: Front Oncol. 2022 Aug 12;12:882061. doi: 10.3389/fonc.2022.882061 (PMC9413194; doi:10.3389/fonc.2022.882061)

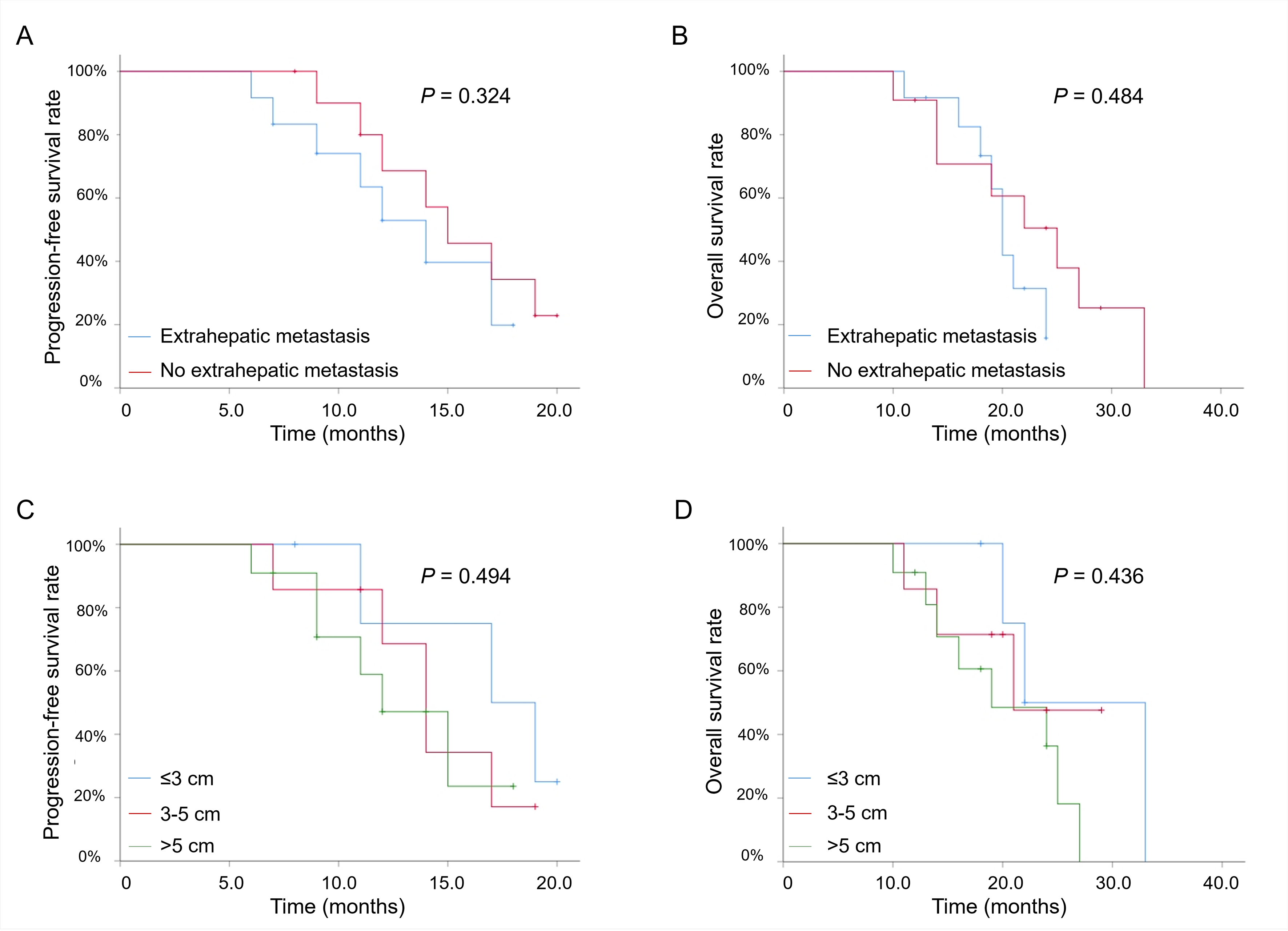

Supplement: Supplementary Figure 1 — Correlation of extrahepatic metastasis with PFS (A) and Correlation of extrahepatic metastasis and tumor size with survival. OS (B); association of tumor size with PFS (C) and OS (D). PFS, progression-free survival; OS, overall survival. [file Image_1.tif]
