# Supplementary material for: CalliSpheres® microsphere transarterial chemoembolization combined with 125I brachytherapy for patients with non–small‐cell lung cancer liver metastases
Source: Front Oncol. 2022 Aug 12;12:882061. doi: 10.3389/fonc.2022.882061 (PMC9413194; doi:10.3389/fonc.2022.882061)
Supplement: Supplementary file 2 [file Table_1.docx]

Supplementary Table 1. Major complications of ^125^I

| Items | No. (%) |
| --- | --- |
| Intrahepatic bile duct injury | 1 (4.3) |
| Vascular Injury | 2 (8.7) |
| Pneumothorax | 1 (4.3) |
